# Fistules obstétricales dans la province du Haut-Katanga, République Démocratique du Congo: à propos de 242 cas

**DOI:** 10.11604/pamj.2018.29.34.14576

**Published:** 2018-01-16

**Authors:** Joseph Bulanda Nsambi, Olivier Mukuku, Jean-de-Dieu Foma Yunga, Xavier Kinenkinda, Prosper Kakudji, Justin Kizonde, Jean-Baptiste Kakoma

**Affiliations:** 1 Département de Gynécoloie-Obstétrique, Faculté de Médecine, Université de Lubumbashi, Lubumbashi, République Démocratique du Congo; 2 Institut Supérieur des Techniques Médicales, Lubumbashi, République Démocratique du Congo; 3 Institut Supérieur des Techniques Médicales, Uvira, République Démocratique du Congo

**Keywords:** Fistule obstétricale, prise en charge, Haut-Katanga, République Démocratique du Congo, Obstetric fistula, treatment, northern Katanga province, Democratic Republic of the Congo

## Abstract

Les fistules obstétricales constituent un problème majeur de Santé Publique dans les pays pauvres. L’objectif de ce travail est de décrire les aspects épidémiologiques, cliniques et thérapeutiques des fistules obstétricales dans la province du Haut-Katanga, République Démocratique du Congo. L’étude est transversale descriptive, portant sur 242 patientes souffrant de la fistule obstétricale dans la province du Haut-Katanga durant la période allant de Septembre 2009 à Décembre 2013. Les paramètres étudiés étaient les données sociodémographiques maternelles, les paramètres obstétricaux et néonatals, les caractéristiques spécifiques de la fistule ainsi que les modalités et l’issue de la prise en charge. Les variables ont été analysées sur logiciel Epi-info version 7.1. Des 242 cas de fistules, 229 patientes ont accouché par voie basse soit 95% d’entre elles et 74,6% à domicile. Le nouveau-né était décédé en période périnatale dans 93,4% des accouchements. L’âge moyen des patientes était de 27,9±10,3 ans. Environ une patiente sur six avait moins de 20 ans et dans l’ensemble près d’une patiente sur 2 avait moins de 25 ans. 7 patientes sur 10 avaient une parité inférieure à 3 et la parité moyenne était de 2,5±2,0. Nonante pourcent des cas avaient un niveau d’études bas et 95% vivaient seules. La fistule avait en moyenne plus de 4,7±4,4 ans d’âge, était vésico-vaginale (96%), de type 2-3 (37%) et réparée par voie vaginale (67%). Le taux d’échecs était de 14%. La fistule obstétricale constitue un réel problème de Santé Publique dans notre milieu et mérite une réflexion profonde pour son éradication.

## Abstract (en)

Obstetric fistulas are a major public health problem in poor countries. This study aimed to describe the epidemiological, clinical and therapeutic features of obstetric fistulas in northern Katanga province, Democratic Republic of the Congo. We conducted a descriptive cross-sectional study of 242 patients with obstetric fistula living in northern Katanga province from September 2009 to December 2013. The parameters studied included maternal sociodemographic data, obstetric and neonatal parameters, specific features of the fistula as well as management approach and outcome. Variables were analyzed using Epi Info 7.1 software. Out of 242 patients with obstetric fistula, 229 (95%) patients delivered vaginally and among them 74.6% delivered at home. In 93,4% of cases the new-born died in the perinatal period. The average age of patients was 27.9 ± 10.3 years. One out of six patients had less than 20 years and in general almost 1 out of 2 patients had less than 25 years. Seven out of ten patients had a parity of less than 3 and the average parity was 2.5±2.0. Ninety percent of patients had a low educational level and 95% lived alone. The patients had, on average, a history of fistula of more than 4.7±4.4 years, it was a vesico-vaginal fistula (96%) type 2-3 (37%) and it was repaired using a transvaginal approach (67%). The average failure rate was 14%. Obstetric fistula is a real public health problem in our environment and it deserves further consideration to be eradicated.

## Introduction

Les fistules urogénitales (FUG) se définissent comme une communication anormale, congénitale ou acquise entre l’appareil urinaire et l’appareil génital féminin. Cette communication peut apparaître entre la vessie et l’utérus, la vessie ou l’urètre et le vagin. On distingue ainsi diverses variétés de FUG: les fistules urétro-vaginales, les fistules vésico-utérines et les fistules vésico-vaginales. De nombreuses étiologies sont reconnues aux FUG, mais deux d’entre elles caractérisent cette pathologie en Afrique: l’étiologie obstétricale et l’étiologie traumatique. Dans les pays nantis, les fistules sont dues à des causes iatrogènes, généralement suite à une radiothérapie et des interventions chirurgicales [1]. Dans les pays à faibles ressources où l’accès aux soins pendant l’accouchement reste restreint, les fistules sont dues à un travail prolongé ou dystocique, survenant le plus souvent lorsque la tête du fœtus se coince dans le petit bassin de la mère coupant ainsi le flux sanguin vers les tissus environnants. Cette ischémie prolongée peut évoluer vers la nécrose tissulaire dont la chute conduit à la formation de la fistule [2]. Des études réalisées en République Démocratique du Congo (RDC) et ailleurs en Afrique ont montré que, dans la majorité des cas, les FUG obstétricales surviennent à la suite des dystocies caractérisées par un travail prolongé sans possibilité de césarienne [3]. Ce fait est en relation directe avec la sous-médicalisation, la pauvreté et la situation de guerre en RDC en particulier et en Afrique subsaharienne en général [3,4]. L’incidence exacte des fistules reste difficile à apprécier car les données statistiques concernant l’ampleur de l’affection sont difficiles à déterminer avec certitude compte tenu de l’absence d’enquêtes épidémiologiques appropriées. L’Organisation Mondiale de la Santé (OMS) estime que plus de 2 millions de jeunes femmes à travers le monde vivent avec une fistule non traitée et que 50.000 à 100.000 nouvelles femmes sont touchées chaque année. La grande majorité des cas est recensée en Afrique subsaharienne et en Asie du Sud-Est [5,6]. En RDC, l’Enquête Démographique et de Santé (EDS) de 2007 indique que 0,3% des femmes déclarent avoir déjà présenté des symptômes de la fistule [7]. Les FUG constituent ainsi un problème majeur de Santé Publique dans les pays pauvres. Les fistules urogénitales sont un fléau persistant malgré les campagnes de préventions et d’éradication comme le constate le rapport d’évaluation du Fonds des Nations Unies pour la Population en 2003 [8]. Les femmes avec fistules vivent souvent dans des conditions difficiles associées qui découlent soit de la fistule elle-même soit du travail prolongé [9]. Les conséquences les plus évidentes sont l´incontinence, soit urinaire, soit des matières fécales ou soit encore des deux [9]. La fuite constante d´urines et de fèces peut également entraîner des dommages à la vulve et les cuisses [10]. Les fistules conduisent à l’ostracisme social et à la stigmatisation [11]. Beaucoup de séries de cas montrent des taux élevés de divorce ou de séparation conjugale, l’absence de rapports sexuels, la perte de fertilité, l’aménorrhée et la dépression chez les femmes fistuleuses [11-13]. Les FUG affectent dans la majorité des cas des femmes jeunes. Celles-ci sont le plus souvent à leur première grossesse qui, à l’issue d’une dystocie, débouche sur la mort du fœtus [9]. Le présent travail s’est fixé comme objectif de décrire les aspects épidémiologiques, cliniques et thérapeutiques des FUG d’origine obstétricale tels qu’ils sont actuellement observés dans la province du Haut-Katanga en RDC.

## Méthodes

### Cadre d’étude

**Figure 1:**
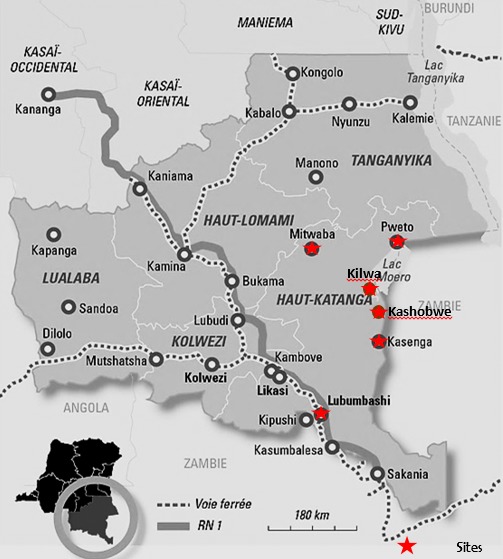
Cartographie du cadre d’étude

Le Katanga est la province la plus méridionale de la République Démocratique du Congo, dont le chef-lieu est Lubumbashi. Sa superficie est de 497 000 km^2^. Ce travail est le fruit de 5 campagnes d’opérations gratuites des FUG organisées par les Organisations non gouvernementales (UNFPA, Médecins du Désert, Médecins sans Frontières/Hollande) en collaboration avec le ministère provincial de la Santé Publique, Province du Katanga qui avaient voulu donner accès aux soins spécialisés à la population de ladite province plus précisément celle habitant dans les zones de santé suivantes: Pweto, Kilwa, Mitwaba, Kasenga, Kashobwe et Lubumbashi (Figure 1). Cette activité a été réalisée dans les hôpitaux généraux de référence de Kashobwe, Pweto, Mitwaba, Kilwa, Dubié et l’hôpital Gecamines/Sud à Lubumbashi. Les hôpitaux concernés étaient choisis pour leur accessibilité géographique et pour leur plateau technique afin de rendre possible un grand nombre d’interventions chirurgicales. L´équipe opératoire était composée de deux chirurgiens, d´un anesthésiste et de deux infirmiers.

### Type et période d’étude

Il s’agit d’une étude transversale descriptive. Elle s’est déroulée de Septembre 2009 à Décembre 2013.

### Population d’étude

L’étude a porté sur un effectif de 242 femmes porteuses de FUG d’origine obstétricale (FO) venues après sensibilisation communautaire dans les villes et les villages précités ainsi que dans leurs environs. Le recueil des données s’est fait grâce à un questionnaire dont les données ont été recueillies à partir de l’interrogatoire des patientes, des registres de consultations externes, du registre des salles d’opération et des registres d’hospitalisation. Ces documents ont permis d’avoir des renseignements nécessaires sur les femmes depuis leur admission dans le service jusqu’à leur sortie.

*Critères d’inclusion:* Toute femme porteuse d’une FO reçue en consultation externe ou référée, et qui a bénéficié d’une prise en charge chirurgicale au cours de la période d’étude et dans notre cadre d’étude.

*Mode de recrutement:* Ce mode de recrutement consiste à des périodes de sensibilisation par des Organisations Non Gouvernementales (ONG) œuvrant dans ce sens. Elles vont jusqu’aux confins du pays chercher les patientes. Certaines nous ont été adressées par les structures sanitaires de l’intérieur du pays.

### Variables d’étude

*Caractéristiques sociodémographiques, anthropométriques et gynéco-obstétricales:* Age (lors de la prise en charge et lors de la survenue de la fistule), parité (lors de la prise en charge et lors de la survenue de la fistule), nombre d’enfants décédés dans la progéniture de la patiente, niveau de scolarité, lieu d’accouchement (à domicile, dans un centre de santé ou dans un hôpital général de référence), issue du nouveau-né (décédé ou vivant), situation maritale actuelle (vivant seule ou vivant en couple), durée de travail lors de la survenue de la fistule, antécédent de césarienne, taille, poids et indice de masse corporelle.

*Paramètres en rapport avec la fistule:* Age de la fistule, circonstance de survenue de la fistule (lors de l’accouchement par voie basse ou lors de l’accouchement par césarienne après échec de la voie basse), taille de la fistule et type de fistule. Les fistules ont été classées selon la classification de Goh [14] en types 1, 2, 3 et 4.

*Aspects thérapeutiques:* résultat de la cure (succès lorsque la fistule est fermée ou échec lorsque la fistule n’est pas fermée), voie d’abord (voie basse, voie haute ou voie mixte), nombre de cures antérieures de la fistule.

***Critères d’éligibilité à la chirurgie:*** Vu le plateau technique et la courte période des campagnes, les femmes prises en charge étaient celles porteuses d’une fistule simple (ou type 1) ou d’une fistule complexe de type 2 ou 3 selon la classification de Goh. Toute femme porteuse d’une fistule complexe de type 4 ou fistule grave (transsection) n’avait pas été retenue.

***Intervention chirurgicale:*** Toutes les interventions chirurgicales ont été pratiquées dans les blocs opératoires. En moyenne quatre patientes étaient opérées par journée opératoire. La technique chirurgicale était fonction du type de FUG et de l’expérience de l’opérateur. La voie basse et la technique classique de Chassar-Moir ont été les plus utilisées. Durant les opérations, les chirurgiens étaient assistés par des médecins généralistes.

## Résultats

### Caractéristiques des fistuleuses lors de la cure

L’âge moyen des patientes lors de la cure de la fistule était de 27,96 ± 10,37 ans (extrêmes: 14 et 68 ans) et la tranche d’âge compris entre 20 et 24 ans était la plus concernée (71 cas soit 29,3%). Nous avons noté que 46,2% des patientes avaient un âge inférieur à 25 ans. La moyenne de la parité était de 2,5 ± 2,0 (extrêmes: 1 et 12) et près de 2/5 des patientes étaient à leur première expérience de maternité. La taille moyenne des patientes était de 145,1±7,1 cm (extrêmes: 135 et 164 cm) et près de ¾ des patientes avaient une taille inférieure à 150 cm. Le poids moyen des patientes était de 46,7 ± 6,5 kg (extrêmes: 34 et 68 kg) et près de 7 malades sur 10 avaient un poids inférieur à 50 kg. L’IMC moyen des patientes était de 22,1 ± 2,1 kg/m^2^(extrêmes: 15,3 et 28,4 kg/m^2^); 4,1% d’entre elles étaient dénutries et 7,9% étaient en surpoids. Des 242 patientes, près de 70% avaient eu une scolarité de niveau primaire. En ce qui concerne le contexte conjugal, près de 4/5 des patientes vivaient seules lors de la prise en charge. Près de 20% des patientes étaient césarisées au moins une fois et 34 malades (14%) avaient déjà bénéficié d’au moins une cure de la fistule dont 26 (76%) une fois (Tableau 1).

**Tableau 1:** Caractéristiques des patientes lors de la fistulorraphie

Variable	Effectif	Pourcentage
**Âge**		
< 20 ans	41	16,9
20-24 ans	71	29,3
25-29 ans	49	20,2
30-34 ans	30	12,4
35-39 ans	20	8,3
≥ 40 ans	31	12,8
**Parité**		
1	103	42,6
2	64	26,4
3-4	34	14,0
≥ 5	41	16,9
**Taille**		
< 150 cm	177	73,2
≥ 150 cm	65	26,8
**Poids**		
< 50 kg	165	68,2
≥ 50 kg	77	31,8
**Indice de masse corporelle**		
< 18,5 kg/m²	10	4,1
18,5 – 24,9 kg/m²	213	88,0
≥ 25,0 kg/m²	19	7,9
**Niveau de scolarité**		
Non scolarisée	62	25,6
Primaire	167	69,0
Secondaire	13	5,4
**Situation maritale actuelle**		
Seule	173	71,5
En couple	69	28,5
**Fistulorraphies antérieures**		
0	208	86,0
1	26	10,6
2	4	1,7
≥3	4	1,7
**Antécédent de césarienne**		
Aucun	199	82,2
Césarisée une fois	38	15,7
Césarisée deux fois	5	2,1

### Caractéristiques sociodémographiques des fistuleuses lors de la survenue de la fistule

L’âge moyen des patientes lors de la survenue de la fistule était de 23,20 ± 7,72 ans (extrêmes: 13 et 51 ans); 158 des 242 fistuleuses (65,3%) avaient alors un âge inférieur à 25 ans et 61% un âge inférieur à 20 ans. Les malades avaient une parité variant entre 1 et 6 pour une parité moyenne de 1,14 ± 0,57 (extrêmes: 1 et 6) et 90,9% d’ entre elles étaient primipares. Pour l’ensemble des fistules, 94,6% des cas étaient survenus lors d’un accouchement par voie basse. La moyenne de la durée du travail lors de la survenue de la fistule était de 54,0 ± 17,5 heures (extrêmes: 12 et 120 heures). 212 fistuleuses des 242 (87,6%) avaient déclaré avoir connu une parturition supérieure à 24 heures et 86 (40,5%) avaient signalé une durée excédant 48 heures. 70% des patientes avaient accouché à domicile et 93,4% de leurs nouveau-nés étaient décédés en période périnatale (Tableau 2).

**Tableau 2:** Caractéristiques sociodémographiques des patientes lors de la survenue de la fistule

Variable	Effectif(n=242)	Pourcentage
Âge lors de la fistule		
< 20 ans	97	40,1
20-29 ans	103	42,6
≥ 30ans	42	17,3
Parité lors de la fistule		
1	220	90,9
2	14	5,8
≥ 3	8	3,3
Circonstance de survenue de la fistule		
Accouchement par voie basse	229	94,6
Césarienne	13	5,4
Durée du travail d’accouchement		
12 heures	1	0,4
24 heures	29	12,0
36 heures	4	1,7
≥48 heures	208	85,9
Lieu d’accouchement		
A domicile	171	70,7
Centre de santé	48	21,6
Hôpital général de référence	23	10,4
Issue du nouveau-né		
Décédé	226	93,4
Vivant	16	6,6

### Paramètres en rapport avec la fistule

L’âge moyen de la fistule était de 4,97 ± 4,76 ans (extrêmes: 6 mois et 34 ans) et 33,5% des patientes portaient leur fistule depuis au moins 5 ans. Des 242 fistules, 219 (90,5%) étaient de grade inférieur à 3 dont 131 (60%) de type 1. La fistule était de type 4 dans 8,3% et de type 3 dans 1,2% des cas. Quant à la variété anatomique, 96% des fistules étaient vésico-vaginales et 2,1% de nature recto-vésico-vaginale. Le nombre moyen de fistules était de 1,06 ± 0,27 (extrêmes: 1 et 3) et près de 95% des patientes avaient une seule fistule. La taille moyenne des fistules était de 2,47 ± 1,46 cm (extrêmes: 0,5 et 7,0 cm) et 197 fistules (81,4%) mesuraient au plus 3 cm et 25,3% d’ entre elles avaient moins de 1,5 cm. Des 242 fistuleuses enregistrées, 222 avaient bénéficié d’un traitement chirurgical (91,7 %) 20 (8,3 %) n’avaient pas été retenues car le plateau technique ne permettait pas leur prise en charge; il s’était agi des fistules de type 4. Près de 7 fistules sur 10 avaient été réparées par voie vaginale, 25,2% par un abord abdominal et 5,4% par un abord mixte. La cure de la fistule s’était soldée par un échec dans 14% des cas (Tableau 3).

**Tableau 3:** Paramètres en rapport avec la fistule

Variable	Effectif(n=242)	Pourcentage
**Âge de la fistule**		
< 1 an	1	0,4
1 an	44	18,2
2-4 ans	116	47,9
≥ 5 ans	81	33,5
**Type de fistule**		
1	131	54,1
2	88	36,4
3	3	1,2
4	20	8,3
**Variété de fistule**		
Vésico-vaginale	233	96,3
Recto-vésico-vaginale	5	2,1
Recto-vaginale	2	0,8
Urétéro-vaginale	1	0,4
Vésico-utérine	1	0,4
**Nombre de fistules**		
1	229	94,7
2	11	4,5
3	2	0,8
**Taille de la fistule**		
< 1,5 cm	50	20,7
1,5-3 cm	147	60,7
Ø 3 cm	45	18,6
**Voie d'abord^+^**		
Voie basse	154	69,4
Voie haute	56	25,2
Voie mixte	12	5,4
Résultat de la cure		
Succès	191	78,9
Echec	31	12,8
Non opérées	20	8,3
*^+^n = 222*		

## Discussion

### Âge des patientes

L’âge moyen des fistuleuses lors de la cure de la fistule était de 27,9 ans et 46,2% d’entre elles étaient âgées de moins de 25 ans, 16,9% étant des adolescentes. Lors de la survenue de la fistule, cet âge moyen était de 23,2 ans et plus de 65% de fistuleuses étaient âgées de moins de 25 ans, 40,1% étant des adolescentes. Dans l’étude de Harouna, près de 52% des fistuleuses étaient âgées de moins de 20 ans lors de leur prise en charge (âge moyen 19 ans) [15]. En Zambie, Holme avait trouvé que l’âge de fistuleuses lors de la survenue de la fistule était de 22 ans [12]. La fistule obstétricale survient de manière prédilectionnelle chez la jeune parturiente; en effet, le très jeune âge de la patiente a été noté par plusieurs auteurs [12,15]. Des études antérieures ont trouvé un taux élevé de complications obstétricales chez les adolescentes. Tebeu, dans une revue de littérature, a constaté que 8,9 à 86% des fistuleuses étaient adolescentes lors de leur prise en charge [14]. Le risque obstétrical élevé chez les adolescents peut être partiellement expliqué par l´immaturité anatomique. Chez l’adolescente, le bassin croit plus lentement et progressivement jusqu’à l’âge avancé. De plus l’acquisition de la taille adulte n’implique pas une croissance équivalente du bassin car « le bassin ne termine définitivement sa configuration que vers la 25^ème^ année bien que les formes adultes sont atteintes vers l’âge de 16 ans » [16]. Cette immaturité du bassin est responsable des anomalies du bassin (bassin limite, bassin généralement rétréci) chez l’adolescente. Et ce dernier est à son tour responsable des complications obstétricales plus fréquentes [17]. En dehors des pathologies anatomiques du bassin, il existe des pathologies fœtales (de volume, de présentation) conduisant à un défaut d’engagement qui, associé à l’absence de surveillance qualifiée de la parturition et à l’absence de contre-indication de la voie basse, serait également à l’origine de fistules. La fistule est plus l’apanage des femmes rurales qu’urbaines à cause de la disparité qualitative des structures sanitaires concernant notamment la qualification de son personnel. A cela s’ajoutent les habitudes ethnoculturelles qui sacralisent les mariages précoces, les accouchements à domicile et la tentation de tout obtenir par voie basse seule jugée honorable alors que la césarienne revêt un caractère injurieux pour la femme dans notre milieu.

### Parité des patientes

L’étude rapporte que 42,6% des patientes étaient primipares lors de la prise en charge. Au moment de la survenue de la fistule, 90,9% des patientes étaient nullipares. Tebeu a trouvé que 31 à 66,7% des fistuleuses étaient primipares lors de leur prise en charge [14]. Dekou, dans son étude menée en Côte d’Ivoire, a rapporté 44,28% des primipares [18]. Ainsi, cette prédominance des primipares semble être en accord avec certaines données de la littérature. Plusieurs auteurs s’accordent à dire que la majorité des FO surviennent chez les adolescentes primipares du fait d’une disproportion céphalo-pelvienne et d’un travail d’accouchement prolongé [12,19,20].

### Statut matrimonial

L’étude rapporte que 71,5% des patientes vivaient seules au cours de leur maladie. Elles sont abandonnées à leur triste sort, incapables le plus souvent de faire face aux dépenses de soins. Ce constat est similaire aux résultats des études antérieures qui ont révélé que les femmes atteintes d’une fistule obstétrique étaient négligées et abandonnées par leurs maris [21]. Par contre, d’autres ont rapporté des taux faibles des femmes divorcées ou abandonnées: 17,8% pour Jokhio (au Pakistan) [19], 19% pour Washington (au Rwanda) [22], 32,4% pour Kaboré (au Burkina Faso) [23]. Cette différence dans la proportion des femmes mariées est difficile à expliquer, mais pourrait être liée aux différences de culture et de croyances religieuses dans les différentes études.

### Niveau d´instruction

La grande majorité de nos patientes étaient sans aucun niveau d´instruction (94,7%). Ce constat est également fait par la plupart des auteurs africains ayant publié sur les fistules urogénitales: 90,5% des fistuleuses étaient non scolarisées dans l’étude de Jokhio [19] et 92,5% dans celle de Kaboré [23]. Dans une étude zambienne menée par Holme, le niveau d’instruction des fistuleuses était statistiquement très bas comparativement à celui des femmes sans fistule [12]. Le niveau d´instruction étant un reflet indirect du niveau socio-économique, il apparaît que le bas niveau socio-économique et le faible niveau d’instruction sont des caractéristiques de la fistuleuse et ce constat est aussi celui unanimement rapporté par la plupart des auteurs [14,23]. Les femmes sans instruction ou peu instruites sont souvent privées de l’information nécessaire sur l’importance des consultations prénatales et de l’accouchement à l’hôpital; elles n’ont pas souvent aussi accès aux soins de qualité, le niveau d’instruction bas étant souvent en rapport avec le bas niveau socio-économique [14,23]. De ce constat, l’une des stratégies pouvant permettre de prévenir cette affection est l’amélioration du niveau socio-économique et l’élévation du niveau d´instruction de l´ensemble de la population tout en pourvoyant les milieux ruraux des structures sanitaires adéquates avec un personnel qualifié puisque les fistules urogénitales de cause obstétricale ont quasiment disparu dans les pays du Nord avec l’amélioration des conditions de vie et des conditions socio-sanitaires.

### Caractéristiques anthropométriques

La moyenne de poids chez nos patientes était de 46,7±6,5kg et 68,2% des patientes avaient un poids inférieur à 50kg. Pour Wall, le poids moyen était de 43,6kg et 55% des fistuleuses pesaient moins de 50kg [10]. La moyenne de taille chez nos patientes était de 145,1±7,1cm et 73,2% des patientes mesuraient moins de 150 cm. La taille moyenne dans la série de Holme était de 148cm [12]. La petite taille et le déficit pondéral chez les fistuleuses ont été rapportés dans la plupart des séries publiées [10,12]. Ce déficit staturo-pondéral rapporté par les différentes études serait dû à l’anémie, à la malnutrition et aux grossesses précoces survenant avant la fin de la puberté arrêtant précocement la croissance. Selon Lansac, la petite taille constitue un facteur de dystocie classiquement connu [24]. La discussion aurait été plus aisée à ce sujet si l’on avait pris soins d’évaluer le bassin obstétrical des fistuleuses. Dans les milieux où nous avons mené notre étude, le mariage a lieu traditionnellement à un âge précoce. Puisque la croissance de la taille s’arrête typiquement à la survenue des premières règles, alors que la croissance de la capacité pelvienne continue [25], des femmes qui sont mariées très tôt sont exposées au risque de grossesse avant qu’elles aient atteint leur pleine capacité pelvienne adulte. Les femmes de cette série étaient minces et courtes, deux facteurs de risque indépendants pour le travail dystocique [26]. Les études comparatives sur la pelvimétrie ont suggéré que les bassins des femmes africaines seraient plus étroits que ceux de leurs homologues européennes [27]. Ainsi, la combinaison du mariage précoce, de la petite taille, du déficit pondéral, de la croissance pelvienne inachevée et d’un bassin généralement étroit prédispose cette population des femmes à la disproportion céphalo-pelvienne pendant le travail.

### Environnement obstétrical

Dans notre étude, 70,7% des patientes avaient accouché à domicile. Plusieurs auteurs rapportent des taux élevés de parturition dirigée à domicile: 97,1% pour Hilton [28] et 94,8% pour Meyer [20]. Par contre, les résultats rapportés dans l’étude menée par Washington à Kigali (Rwanda) ont montré que 82% des accouchements générateurs de fistules avaient eu lieu dans un centre de santé [22]. Concernant la durée du travail lors de la survenue de la fistule, la moyenne était de 2,25 jours et 99,6% des patientes avaient passé 24 heures ou plus en travail d’accouchement. Dans la littérature, cette durée moyenne variait de 2,5 à 4 jours [15,20,28] et 72,5 à 95,7% des fistuleuses avaient travaillé pendant 24 heures ou plus [10,12]. Ceci s´expliquerait par la fréquence des accouchements dystociques sur grossesses non suivies, se déroulant en dehors de centre de santé, sans aucune assistance obstétricale. Elle s’expliquerait aussi par le fait que nos patientes, rurales pour la plupart résident dans des contrées reculées, enclavées avec un réseau routier médiocre rendant les évacuations d´urgence pour dystocie difficiles sinon tardives et il faut souvent parcourir des centaines de kilomètres pour atteindre un centre de santé avec une hypothétique antenne chirurgicale fonctionnelle.

Selon Meyer, les raisons de la décision retardée de recourir aux soins pourraient inclure des raisons financières, culturelles, religieuses et géographiques, avec beaucoup de femmes vivant simplement trop loin d’un dispensaire pour recevoir les soins opportuns. Etant donné le niveau de performance médiocre des centres médico-sociaux, il n´est pas étonnant de constater que beaucoup de femmes redoutent de s’y présenter [20]. De même, l’inaccessibilité relative des soins de santé mène des hommes et des femmes nécessitant ces soins à consulter tardivement. Les mauvais résultats enregistrés à la suite d´un retard dans la décision de demander des soins sont souvent perçus par le grand public comme la preuve que les centres de soins de santé sont la cause du problème plutôt qu’une solution [20]. C’est particulièrement vrai avec le travail d’accouchement. Dans notre étude, la plupart des femmes (94,8%) ont commencé leur travail à la maison, sous la surveillance d´un membre de la famille qui n’était pas un préposé qualifié d’accouchement. Quant à l’issue néonatale, plus de 93% des nouveau-nés étaient décédés. Ce taux était de 96% dans l’étude de Holme [12]. Cette surmortalité néonatale se comprend aisément si l´on considère la durée du travail qui est généralement longue pouvant atteindre plusieurs jours et la fistulisation n´est que le résultat de la lutte de l’organisme contre la dystocie. En plus, la population de notre étude a souvent recours à des pratiques particulièrement néfastes lors du travail d’accouchement: pour les accouchements prolongés, on fait pression avec le mortier sur l’abdomen de la parturiente pour faciliter l’expulsion du fœtus.

***Âge de la fistule*** Notre étude relève que 33,5% des patientes portaient leur fistule depuis 5 ans ou plus et l’âge moyen de la fistule était de 4,76±4,97 ans (extrêmes: 6 mois et 34 ans). Au Pakistan, Jokhio a rapporté que 40% des patientes portaient leurs fistules depuis plus de 5 ans [19]. Harouna a rapporté un âge moyen de 19 mois avec des extrêmes de 3 mois et 3 ans et demi [15]. Cet âge avancé de la fistule chez nos patientes peut s’expliquer par plusieurs raisons: 1) Le caractère stigmatisant de la pathologie poussant les malades à l’isolement; 2) La longue tolérance clinique de la maladie puisque n´engageant pas dans l’immédiat le pronostic vital; ceci amène bon nombre de patientes à ne pas consulter préférant vivre leur mal en solitaire; 3) Le manque d’information sur les possibilités de prise en charge chirurgicale de l’affection, obligeant les patientes à recourir au traitement traditionnel; 4) Le manque de moyens financiers pour faire face aux dépenses de soins, qui représente également un facteur pouvant contribuer au retard de consultation; 5) L’absence de politique intégrée de prévention et de prise en charge des fistules obstétricales.L’âge de la fistule est un facteur important dans la prévention des conséquences socioéconomiques et psychologiques. Le long délai d’évolution de la maladie est source de stigmatisation, de discrimination et d’abandon, et est un facteur potentiel de séparation du couple.

### Type de fistule

Dans notre étude, 96,3% des fistules étaient des fistules vésico-vaginales, qui sont les formes anatomiques les plus fréquentes [12,22]. Ce taux était respectivement de 70,6% et 90,9% pour Kaboré [23] et Holme [12]. Cette haute fréquence de la fistule vésico-vaginale par rapport aux autres fistules est probablement due à la plus grande probabilité de compression de la paroi vaginale antérieure par la tête du fœtus contre le bassin osseux entraînant donc plus ischémie de la vessie que le rectum. Les fistules urétéro-vaginales sont rares. Durant une période de 10 ans, Tazi au Maroc a colligé 12 fistules vésico-utérines contre 116 fistules vésico-vaginales [29]. La fréquence rapportée dans la littérature varie entre 6,5 et 8,1% des fistules uro-génitales [30,31]. La fistule urétéro-vaginale est souvent due à une ligature urétérale pendant l’hystérectomie et les fistules vésico-utérines surviennent souvent après césarienne.

### Voie d’abord et résultat de la prise en charge

La voie basse était la plus utilisée dans notre étude. Ceci s’expliquerait non seulement par le fait que les fistules vésico-vaginales sont plus fréquentes que les fistules vésico-utérines qui s’abordent par la voie haute, mais aussi par l’expérience des chirurgiens qui optaient souvent pour la voie basse. Nous avons privilégié personnellement l’approche vaginale lorsque la fistule était près du col de la vessie. Cette voie nous semble la meilleure parce qu’elle donne un bon confort opératoire, un accès direct aux lésions du col et de l’urètre. Les avantages incluent un faible taux de complications, moins de saignement, une récupération post-opératoire rapide et une courte durée d’hospitalisation [32]. Nous réservions l’approche abdominale aux cas dont la fistule ne pouvait pas être correctement visualisée et exposée par voie vaginale, derrière les orifices des uretères, soit à cause de la sténose vaginale, soit dans de rares cas où l’état pathologique intra-abdominal nécessitait des soins simultanés. Elle a cependant un inconvénient, qui est celui de ne pas permettre le repérage des uretères sauf dans les grandes fistules avec perte de substance où on voit tout le reste du plancher vésical [32].

Le taux de guérison était de 86% dans notre série. Nos résultats sont comparables à ceux rapportés par Holme (72,9%) [12]. Le taux de réussite après la fistulorraphie varie d’un centre à un autre et est déterminé par de nombreux facteurs tels que le site de la fistule, le degré de cicatrisation, les tentatives de réparation antérieures, la technique de fistulorraphie et de l´expertise du chirurgien, de l´équipement et des soins infirmiers post-opératoires entre autres.

## Conclusion

Notre étude montre que la fistuleuse est très jeune, primipare, d’un bas niveau d’instruction, vivant seule, ayant connu une parturition non assistée et ayant perdu le fruit de sa grossesse. La fistule avait en moyenne plus de 4 ans d’âge, était vésico-vaginale (96%), de type 2-3 (37%) et réparée par voie vaginale (67%). Le taux d’échec était de 14%. Avec des efforts de campagnes de prévention efficaces, l’augmentation de l’éducation et l’autonomisation des femmes qui subissent déjà les conséquences dévastatrices des dystocies, le nombre des femmes atteintes des fistules obstétricales ainsi que la mortalité maternelle et infantile pourront être amoindris.

### Etat des connaissances actuelles sur le sujet

- Les fistules obstétricales constituent ainsi un problème majeur de Santé Publique dans les pays pauvres;
- Les fistules urogénitales sont un fléau persistant malgré les compagnes de préventions et d’éradication.

### Contribution de notre étude à la connaissance

- A notre connaissance, aucune étude scientifiquement élaborée sur ce sujet n’est disponible dans notre milieu, il était donc impérieux de combler ce vide en y apportant un premier outil de référence;
- Dans notre milieu, la fistuleuse est très jeune, primipare, d’un bas niveau d’instruction, vivant seule, ayant connu une parturition non assistée, ayant perdu le fruit de sa grossesse.

## Conflits d’intérêts

Les auteurs ne déclarent aucun conflit d'intérêts.
